# Forsythoside A and Forsythoside B Contribute to Shuanghuanglian Injection-Induced Pseudoallergic Reactions through the RhoA/ROCK Signaling Pathway

**DOI:** 10.3390/ijms20246266

**Published:** 2019-12-12

**Authors:** Jiayin Han, Yushi Zhang, Chen Pan, Zhong Xian, Chenling Pan, Yong Zhao, Chunying Li, Yan Yi, Lianmei Wang, Jingzhuo Tian, Suyan Liu, Dunfang Wang, Jing Meng, Aihua Liang

**Affiliations:** Key Laboratory of Beijing for Identification and Safety Evaluation of Chinese Medicine, Institute of Chinese Materia Medica, China Academy of Chinese Medical Sciences, Beijing 100700, China; jyhan@icmm.ac.cn (J.H.); yszhang@icmm.ac.cn (Y.Z.); cpan@icmm.ac.cn (C.P.); xianzhonglove@126.com (Z.X.); panchenling@outlook.com (C.P.); yzhao@icmm.ac.cn (Y.Z.); cyli@icmm.ac.cn (C.L.); yyi@icmm.ac.cn (Y.Y.); lmwang@icmm.ac.cn (L.W.); jztian@icmm.ac.cn (J.T.); syliu@icmm.ac.cn (S.L.); wdf122644@126.com (D.W.); icmmmj@163.com (J.M.)

**Keywords:** forsythoside A, forsythoside B, forsythoside E, Shuanghuanglian injection, pseudo-allergic reactions, RhoA/ROCK signaling pathway

## Abstract

In recent years, hypersensitivity reactions to the Shuanghuanglian injection have attracted broad attention. However, the componential chief culprits inducing the reactions and the underlying mechanisms involved have not been completely defined. In this study, we used a combination of approaches based on the mouse model, human umbilical vein endothelial cell monolayer, real-time cellular monitoring, immunoblot analysis, pharmacological inhibition, and molecular docking. We demonstrated that forsythoside A and forsythoside B contributed to Shuanghuanglian injection-induced pseudoallergic reactions through activation of the RhoA/ROCK signaling pathway. Forsythoside A and forsythoside B could trigger dose-dependent vascular leakage in mice. Moreover, forsythoside A and forsythoside B slightly elicited mast cell degranulation. Correspondingly, treatment with forsythoside A and forsythoside B disrupted the endothelial barrier and augmented the expression of GTP-RhoA, p-MYPT1, and p-MLC2 in a concentration-dependent manner. Additionally, the ROCK inhibitor effectively alleviated forsythoside A/forsythoside B-induced hyperpermeability in both the endothelial cells and mice. Similar responses were not observed in the forsythoside E-treated animals and cells. These differences may be related to the potential of the tested compounds to react with RhoA-GTPγS and form stable interactions. This study innovatively revealed that some forsythosides may cause vascular leakage, and therefore, limiting their contents in injections should be considered.

## 1. Introduction

Shuanghuanglian injection (SHLI) is a famous antiviral and antimicrobial Chinese medicine administered through intravenous injection. It is derived from extracts of *Flos lonicerae japonicae*, *Radix scutellariae*, and *Fructus forsythia.* Since 1973, it has been widely used alone or as complementary medicine to treat respiratory tract infections, bronchiolitis, and pneumonia in China. Clinical trials indicate that the SHLI is more effective than common antibiotics in relieving the symptoms and duration of fever, chest wheezes, cough, sore throat, and nasal congestion in respiratory tract infections [1,2,3,4]. However, SHLI is reported to cause adverse drug reactions (ADRs) in approximately 3.25% of patients [5]. More than 70% of cases have been recorded as hypersensitivity reactions, mainly manifested as skin, gastrointestinal, and respiratory system disorders, as well as systemic anaphylaxis and anaphylactic shock [6,7]. Consequently, the Chinese Food and Drug Administration has raised the alarm regarding the ADRs of SHLI. They have specifically detailed the most frequent hypersensitivity reactions in the *China State Food and Drug Administration Annual Adverse Reactions Report for Drugs* from 2011 to 2016. Currently, the adverse reactions of SHLI have emerged as a research hotspot and the use of SHLI is mostly limited.

RhoA acts as a molecular switch that cycles between an active GTP-bound form and an inactive GDP-bound form that responds to cell surface receptors. Activation of Rho-associated kinase (ROCK), an immediate downstream effector of RhoA, upregulates the phosphorylation of myosin light chain (MLC). It also inactivates MLC phosphatase and increases endothelial permeability, which is essential for inflammatory responses [8,9,10,11]. Currently, the RhoA/ROCK signaling pathway has been reported to participate in many stimulus-induced endothelial hyperpermeability reactions [12,13,14,15].

**Figure 1:**
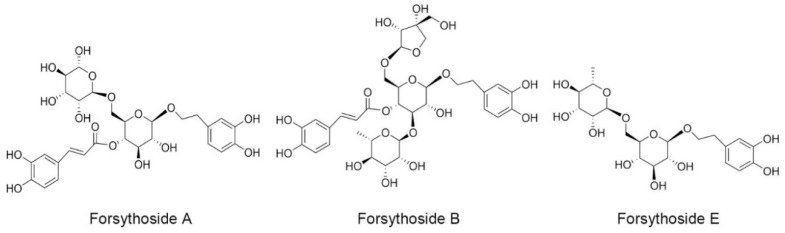
Chemical constructions of forsythoside A, forsythoside B, and forsythoside E.

In our previous study, we demonstrated that SHLI could induce IgE-independent but dose-related pseudoallergic reactions by evoking histamine release, activating the RhoA/ROCK signaling pathway, and triggering increased vascular leakage [16]. This effect, at least partly, results in the hypersensitivity reactions of SHLI in the clinic. In addition, among the extracts of *Flos lonicerae japonicae*, *Radix scutellariae*, and *Fructus forsythia*, we found evidence that the extract of *Fructus forsythia* had the strongest potential to induce pseudoallergic reactions. Meanwhile, when isolated by different polar solvents, the n-butanol extracts of *Fructus forsythia*, especially forsythoside A, were recognized as the main culprits [17]. Forsythoside A is one of the main active phenylethanoid glycosides isolated from the fruits of *Fructus forsythia*. Except for forsythoside A, there have been more than thirty phenylethanoid glycosides isolated from *Fructus forsythia* [18,19], where some are also contained in SHLI [20]. These phenylethanoid glycosides exhibit anti-inflammatory, antioxidant, and antibacterial activities, and so far, no adverse reactions have been observed [18]. Currently, forsythoside A, forsythoside B, and forsythoside E are the most common and available phenylethanoid glycosides reported in SHLI [21] (Figure 1). They were also detected in the SHLI sample at contents of 1.2%, 1.3%, and 1.5%, respectively (Figure S1). In the present study, we investigated the ability of forsythoside A, forsythoside B, and forsythoside E to cause pseudoallergic reactions. Furthermore, we also evaluated whether the effects were associated with the activation of the RhoA/ROCK signaling pathway.

## 2. Results

### 2.1. Forsythoside A and Forsythoside B Induce Vascular Leakage in Mice

**Figure 2:**
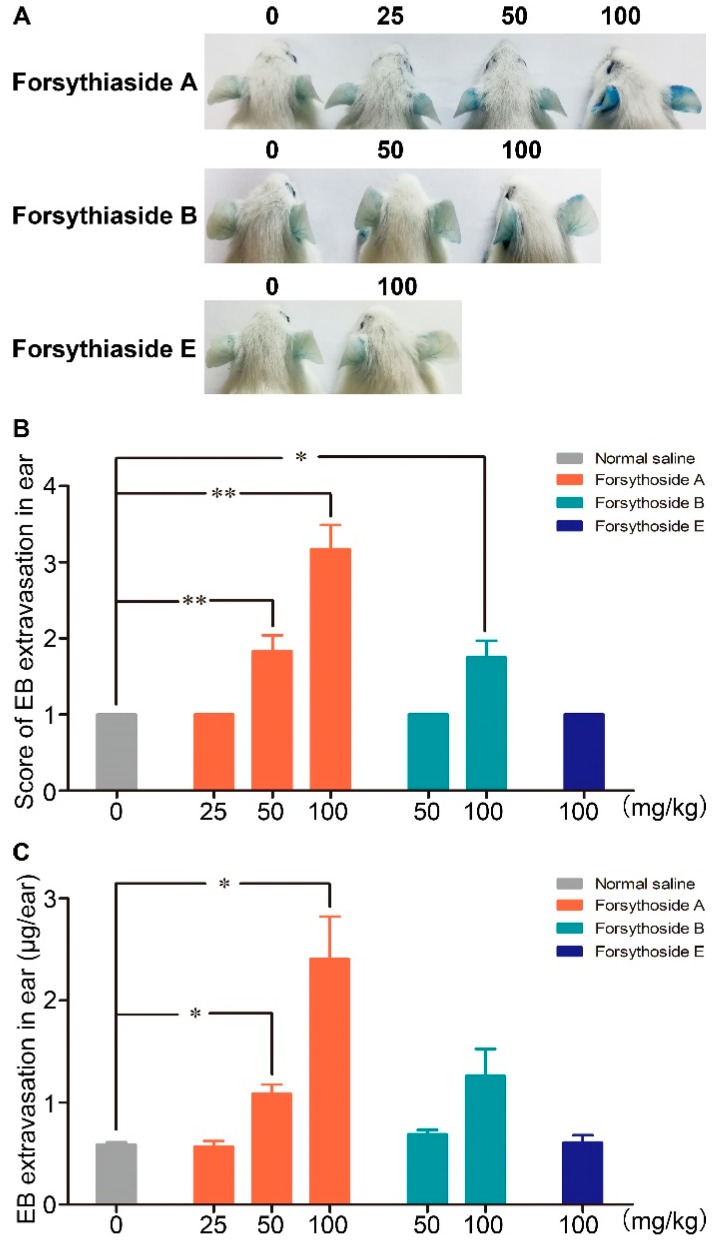
Forsythoside A and forsythoside B induce vascular leakage in mice. (**A**) Vascular leakage in mice injected with forsythoside A/Evans blue (EB), forsythoside B/EB, or forsythoside E/EB. (**B**) The score of blue stain in the ears of mice injected with forsythoside A/EB, forsythoside B/EB, or forsythoside E/EB (*n* = 6). (**C**) Quantification of the EB extracted from the ears of mice injected with forsythoside A/EB, forsythoside B/EB, or forsythoside E/EB (*n* = 6). The same vehicle control group (normal saline) was used for forsythoside A, forsythoside B, and forsythoside E. * *p* < 0.05, ** *p* < 0.01 compared with the normal saline-treated group.

Evans blue (EB), which can strongly bind to albumin and form a stable blue tracer, has been widely applied to evaluate the extent of plasma protein leakage out of blood vessels. In this experiment, the blue stain caused by albumin-EB complex extravasation in the ears of mice was applied as a marker to assess vascular leakage. No visible blue stain was observed in the mice treated with normal saline/EB, which indicated that EB alone did not cause vascular leakage. Dose-dependent EB extravasation occurred in the mice injected with 100 and 50 mg/kg of forsythoside A/EB, respectively, or 100 mg/kg of forsythoside B/EB. The reaction was initially observed in most animals after approximately 10 min, and it became remarkably severe at about 25 min after drug/EB administration. EB extravasation in mice treated with 100 mg/kg of forsythoside A was more severe than that in the animals treated with 100 mg/kg of forsythoside B. In addition, aberrant vascular leakage was observed in the mice injected with 50 mg/kg of forsythoside A, while no apparent reaction was observed in the 50 mg/kg forsythoside B group. Hence, forsythoside A showed greater potential to enhance vascular permeability than forsythoside B. Meanwhile, 100 mg/kg of forsythoside E/EB did not elicit vascular leakage when compared with the control group (Figure 2).

### 2.2. Forsythoside A and Forsythoside B Slightly Evoke Mast Cell Degranulation

**Figure 3:**
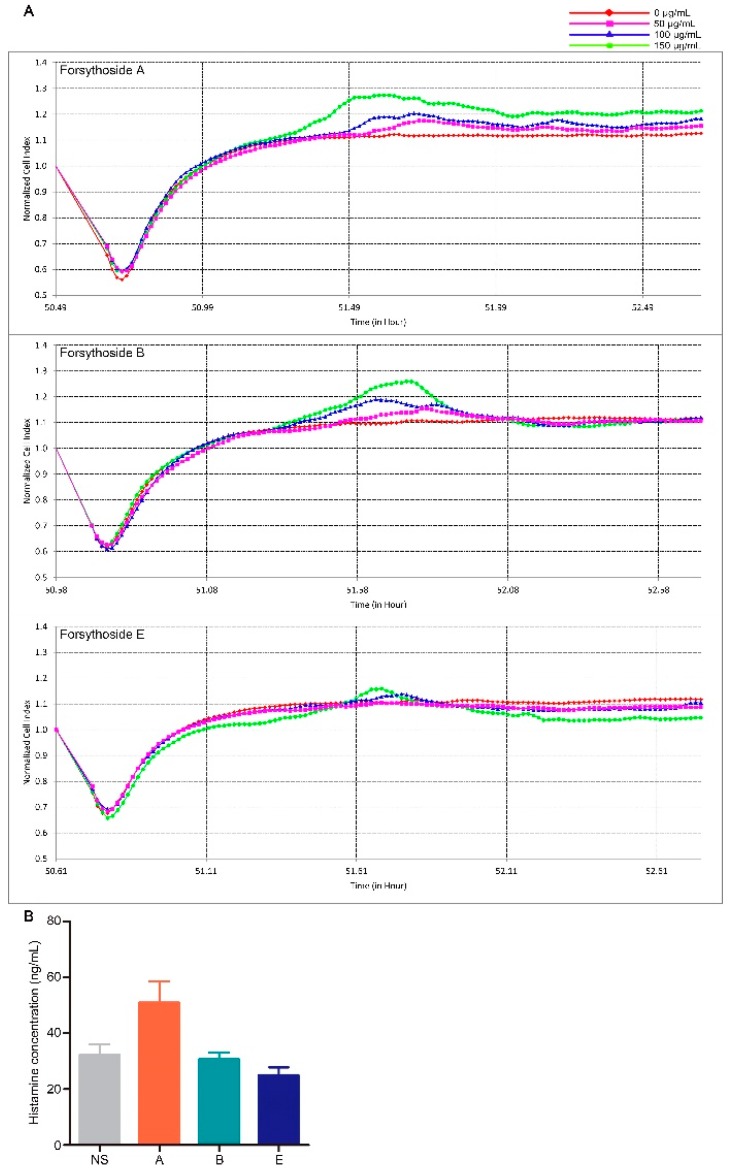
Forsythoside A and forsythoside B slightly evoke mast cell degranulation. (**A**) Cell index of the mast cells incubated with forsythoside A, forsythoside B, or forsythoside E. (**B**) Histamine concentration in the plasma of mice treated with forsythoside A, forsythoside B, and forsythoside E (*n* = 10). (NS: mice treated with normal saline, A: mice treated with forsythoside A, B: mice treated with forsythoside B, E: mice treated with forsythoside E).

Mast cell degranulation associated with histamine release is a principal triggering factor for pseudoallergic reactions [22]. Therefore, we explored whether forsythoside A, forsythoside B, and forsythoside E could evoke this type of reaction. Mast cell degranulation was evaluated according to real-time monitoring of cellular morphology using an RTCA TP instrument [23]. The cell index (CI) value as read on the machine directly responds to the cell attaching properties, which vary when degranulation occurs [24,25]. According to the CI curve, forsythoside A and forsythoside B could stimulate mast cell degranulation. The index was slightly augmented after the RBL-2H3 cells were incubated with forsythoside A and forsythoside B (50, 100, or 150 μg/mL) for about 1 h, as compared with the control group. The changes presented in a concentration-dependent manner. No apparent alterations were observed from the CI curve in the forsythoside E-treated cells (Figure 3A). Histamine release was also estimated from in vivo experiments. Plasma histamine concentration was slightly elevated in the mice treated with forsythoside A at 100 mg/kg, as compared with the control group. Meanwhile, forsythoside B and forsythoside E did not promote histamine release (Figure 3B).

### 2.3. Forsythoside A and Forsythoside B Enhance Endothelial Permeability via Activation of the RhoA/ROCK Signaling Pathway

**Figure 4:**
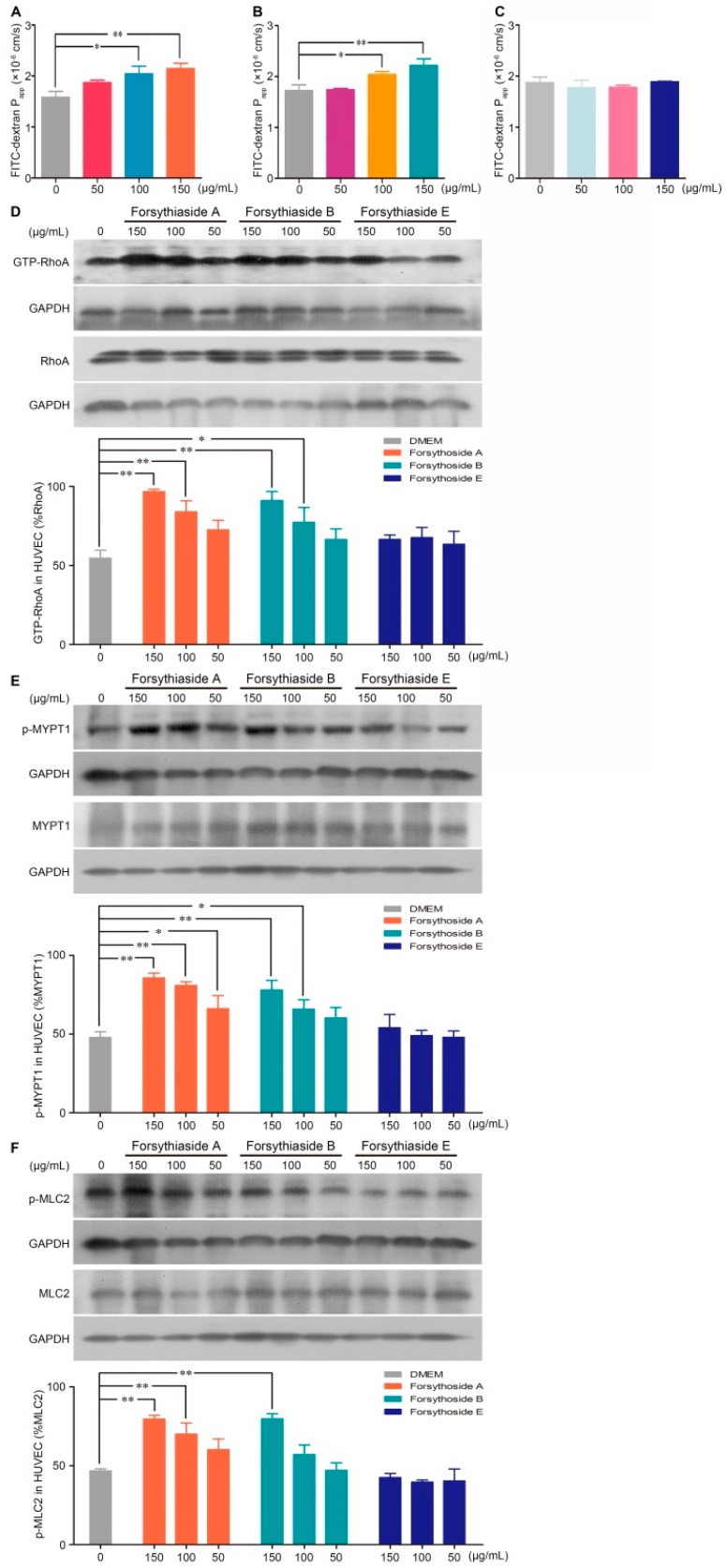
Forsythoside A and forsythoside B alter the endothelial monolayer permeability and expression of GTP-RhoA, p-MYPT1, and p-MLC2 in human umbilical vein endothelial cells (HUVECs). (**A**) Fluorescence apparent permeability coefficient of FITC-dextran through a monolayer of confluent HUVECs incubated with forsythoside A (*n* = 3). (**B**) Fluorescence apparent permeability coefficient of FITC-dextran through a monolayer of confluent HUVECs incubated with forsythoside B (*n* = 3). (**C**) Fluorescence apparent permeability coefficient of FITC-dextran through a monolayer of confluent HUVECs incubated with forsythoside E (*n* = 3). (**D**) Western analysis of GTP-RhoA in HUVECs incubated with forsythoside A, forsythoside B, and forsythoside E (*n* = 3). (**E**) Western analysis of p-MYPT1 in HUVECs incubated with forsythoside A, forsythoside B, and forsythoside E (*n* = 3). (**F**) Western analysis of p-MLC2 in HUVECs incubated with forsythoside A, forsythoside B, and forsythoside E (*n* = 3). The same GAPDH was used for p-MYPT1 and p-MLC2, and the same GAPDH was used for MYPT1 and MLC2, respectively. * *p* < 0.05, ** *p* < 0.01 compared with the DMEM-treated group.

Vascular hyperpermeability is largely determined by the dysfunction of the endothelial barrier. Next, we investigated whether forsythoside A, forsythoside B, and forsythoside E could influence the endothelial cell barrier of blood vessels. By testing the diffusion of FITC-dextran across the intact endothelial monolayer, we found that stimulation with forsythoside A or forsythoside B for 1 h significantly elevated the endothelial monolayer permeability. Forsythoside A and forsythoside B both augmented the permeability coefficient of FITC-dextran in a concentration-dependent manner. Meanwhile, we did not observe a similar endothelial monolayer hyperpermeability in the forsythoside E-stimulated cells, at the tested concentrations (Figure 4A–C).

According to the results of our previous study, the RhoA/ROCK signaling pathway is involved in the SHLI-induced pseudoallergic reactions. Correspondingly, we investigated further whether forsythoside-induced endothelial barrier disruption is linked to RhoA/ROCK signaling pathway activation. The results indicated that forsythoside A and forsythoside B significantly activated the RhoA/ROCK signaling pathway in a concentration-dependent manner. Enhanced expression of GTP-RhoA, p-MYPT1, and p-MLC2 were associated with increased treatment concentrations. No significant upregulation in protein levels was observed in the forsythoside E-treated cells (Figure 4D–F).

### 2.4. Fasudil Attenuates the Forsythoside A/Forsythoside B-induced Endothelial Monolayer and Vascular Hyperpermeability

**Figure 5:**
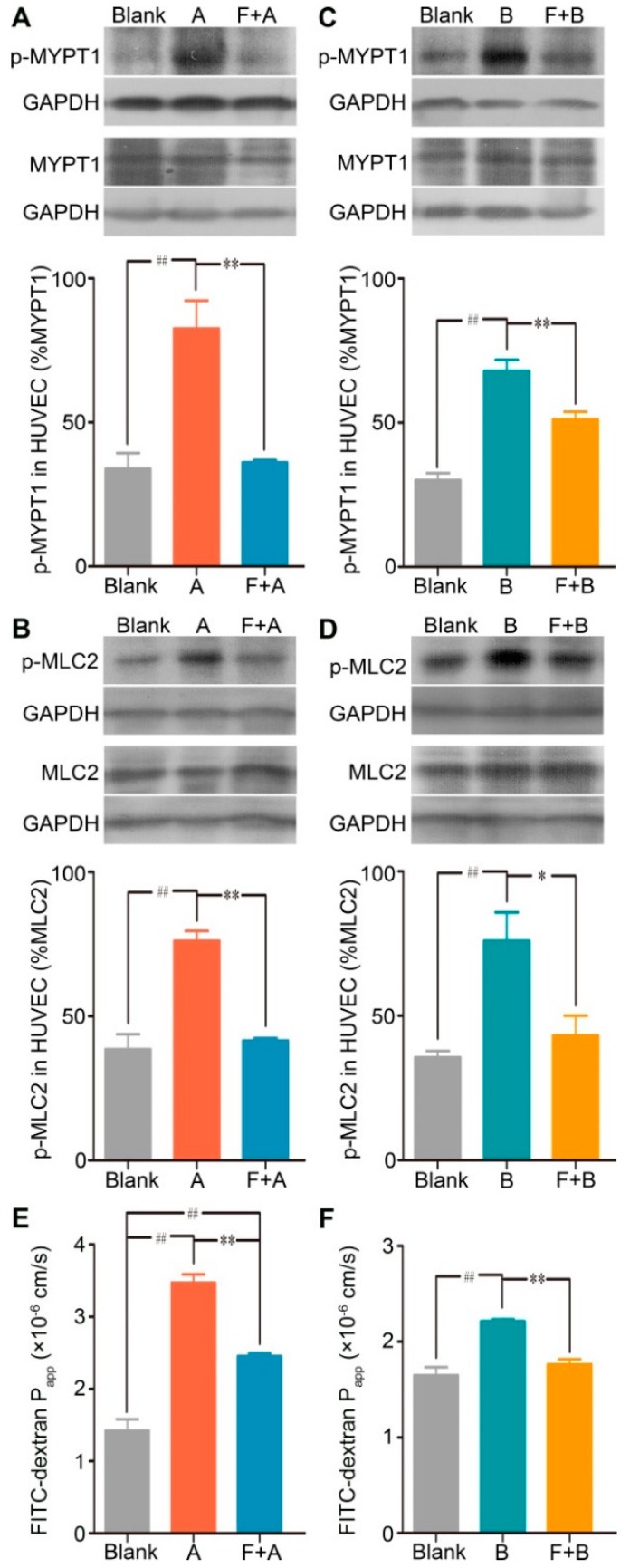
Fasudil hydrochloride suppresses forsythoside A/forsythoside B-induced increased expression of p-MYPT1 and p-MLC2, and it enhances endothelial permeability in HUVECs. (**A**) Western analysis of p-MYPT1 in HUVECs incubated with forsythoside A, with or without fasudil (*n* = 3). (**B**) Western analysis of p-MLC2 in HUVECs incubated with forsythoside A, with or without fasudil (*n* = 3). (**C**) Western analysis of p-MYPT1 in HUVECs incubated with forsythoside B, with or without fasudil (*n* = 3). (**D**) Western analysis of p-MLC2 in HUVECs incubated with forsythoside B, with or without fasudil (*n* = 3). (**E**) Fluorescence apparent permeability coefficient of FITC-dextran through a monolayer of confluent HUVECs incubated with forsythoside A, with or without fasudil (*n* = 3). (**F**) Fluorescence apparent permeability coefficient of FITC-dextran through a monolayer of confluent HUVECs incubated with forsythoside B, with or without fasudil (*n* = 3). * *p* < 0.05, ** *p* < 0.01 compared with the forsythoside A/forsythoside B-treated group. # *p* < 0.05, ## *p* < 0.01 compared with the DMEM-treated group. (Blank: cells treated with culture medium of DMEM, F: cells pretreated with fasudil hydrochloride, A: cells treated with forsythoside A, B: cells treated with forsythoside B).

To further identify the contribution of the RhoA/ROCK signaling pathway in forsythoside A/forsythoside B-induced endothelial barrier dysfunction, we added fasudil hydrochloride, a specific inhibitor of ROCK, to the endothelial monolayer permeability measurement system. As expected, fasudil hydrochloride diminished the forsythoside A/forsythoside B-induced enhancement of endothelial permeability. This outcome was associated with its suppressing effect on the increased expression of p-MYPT1 and p-MLC2 (Figure 5).

**Figure 6:**
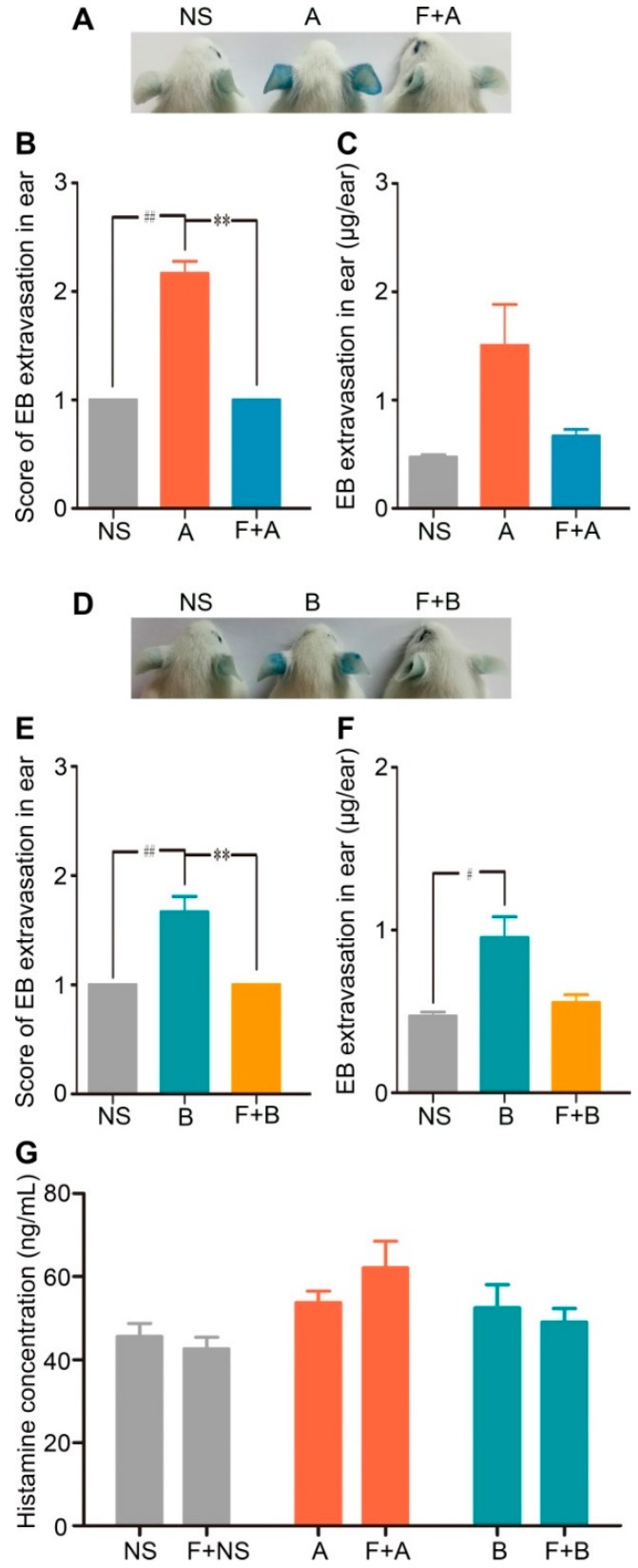
Fasudil hydrochloride inhibits forsythoside A/forsythoside B-induced vascular leakage in mice. (**A**) Vascular leakage in mice injected with forsythoside A, with or without fasudil. (**B**) The score of blue stain in the ears of mice injected with forsythoside A, with or without fasudil (*n* = 6). (**C**) Quantification of the EB extracted from the ears of mice injected with forsythoside A, with or without fasudil (*n* = 6). (**D**) Vascular leakage in mice injected with forsythoside B, with or without fasudil. (**E**) The score of blue stain in the ears of mice injected with forsythoside B, with or without fasudil (*n* = 6). (**F**) Quantification of the EB extracted from the ears of mice injected with forsythoside B, with or without fasudil (*n* = 6). (**G**) Histamine concentration in the plasma of mice treated with forsythoside A and forsythoside B, with or without fasudil (*n* = 10). The same vehicle control group (normal saline) was used for both forsythoside A and forsythoside B. * *p* < 0.05, ** *p* < 0.01 compared with the forsythoside A/forsythoside B-treated group. # *p* < 0.05, ## *p* < 0.01 compared with the normal saline-treated group. (NS: mice treated with normal saline, F: mice pretreated with fasudil hydrochloride, A: mice treated with forsythoside A, B: mice treated with forsythoside B).

Consistent with the results of the in vitro experiment, fasudil hydrochloride prevented the increased vascular leakage evoked by forsythoside A or forsythoside B. When the mice were pretreated with fasudil, the EB extravasation in the ears in response to 100 mg/kg of forsythoside A/EB or forsythoside B/EB was alleviated significantly. Meanwhile, pretreatment with fasudil hydrochloride did not affect histamine release (Figure 6). These results indicated that the RhoA/ROCK signaling pathway contributed to forsythoside A/forsythoside B-induced pseudoallergic reactions independent of the histamine release.

### 2.5. Docking Analysis of GTP-RhoA with Forsythoside A, Forsythoside B, and Forsythoside E

**Figure 7:**
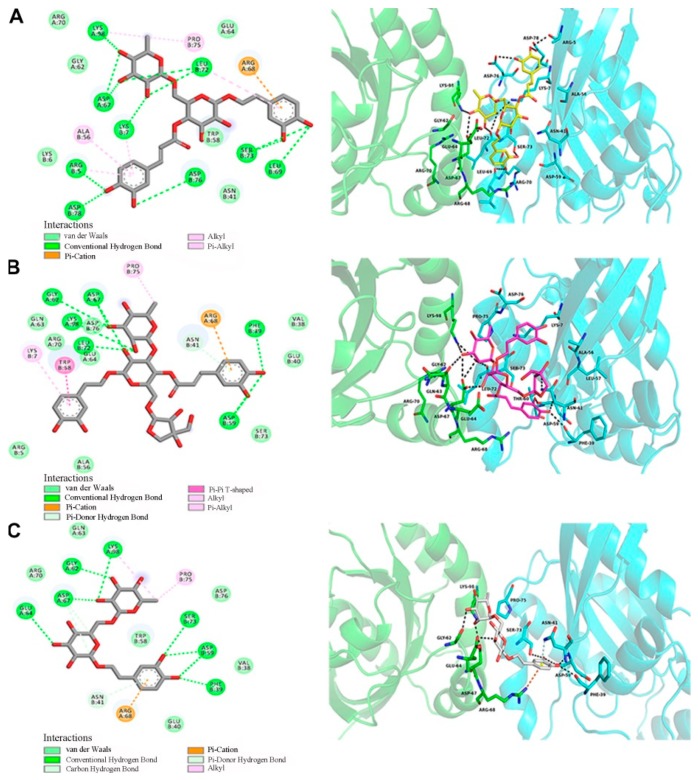
Docking of RhoA-GTPγS with forsythoside A, forsythoside B, or forsythoside E. (**A**) Docking of RhoA-GTPγS with forsythoside A. (**B**) Docking of RhoA-GTPγS with forsythoside B. (**C**) Docking of RhoA-GTPγS with forsythoside E.

To better understand the links between the structures of forsythosides with the RhoA/ROCK signaling pathway, molecular modeling techniques were applied to examine and visualize the compound–protein interactions. Forsythoside A, forsythoside B, and forsythoside E were docked into the pivotal structural regions of RhoA-GTPγS. This was done using an induced fit procedure implemented in the Autodock Vina software (Figure 7). The docking scores of forsythoside A, forsythoside B, and forsythoside E were −7.9, −7.4, and −6.5, respectively. The results revealed that forsythoside A and forsythoside B were more liable to react with RhoA-GTPγS and form more stable interactions, as compared with forsythoside E. This might lead to their different observed capacities to activate the RhoA/ROCK signaling pathway.

## 3. Discussion

In recent years, several studies have been performed to identify the chief componential culprits contributing to SHLI-induced hypersensitivity reactions. Chlorogenic acid [26,27] and baicalin [28,29], contained in SHLI, are commonly reported as being the responsible constituents triggering allergic reactions, due to their antigenic properties. However, based on clinical data, most adverse reactions to SHLI occur at the first exposure [6,30], which might suggest that these reactions are unlikely to be immune-mediated. Some studies have demonstrated that chlorogenic acid can alter the biological characteristics of basophil granulocytes [31]. Chlorogenic acid, cryptochlorogenic acid, ferulic acid, and baicalin can evoke mast cell degranulation and elicit β-hexosaminidase release [32,33,34,35]. In addition, cryptochlorogenic acid and isochlorogenic acid B and C can directly activate blood C5a and provoke histamine release [36]. These results show that some constituents from *Flos lonicerae japonicae* (e.g., chlorogenic acid, cryptochlorogenic acid, isochlorogenic acid B and C, and ferulic acid) and baicalin from *Radix scutellariae* may lead to pseudoallergic hypersensitivity reactions from SHLI. However, these outcomes are still a matter of debate [37,38,39]. In our previous studies, we evaluated the pseudoallergenic potential of the extracts from *Flos lonicerae japonicae*, *Radix scutellariae*, and *Fructus forsythia*. We found that the extracts from *Fructus forsythia* in n-butanol were associated with the pseudoallergic reactions of SHLI. Forsythoside A was recognized as one of the main culprits [17]. In the present study, we demonstrated that forsythoside A and forsythoside B could both activate the RhoA/ROCK signaling pathway and evoke vascular hyperpermeability. This might partly cause the pseudoallergic reactions of SHLI.

The pseudoallergic reactions induced by forsythoside A and forsythoside B were found to be dose-dependent, indicating that the contents of these two constituents might influence the ADRs of SHLI. Currently, forsythoside A is one of the index constituents of *Fructus forsythia* in the Chinese pharmacopeia. Its minimum content in raw medicinal herbs has been clearly defined. However, no maximum limit for content has been set. Meanwhile, forsythoside B, which is recorded as the index constituent of *Callicarpae caulis et folium*, another Chinese medicine with a good effect on hemostatics, also lacks restrictions regarding an upper limit. Besides SHLI, *Fructus forsythia* is also applied in many Chinese medicine injections, such as Qingrejiedu, Sangjiangganmao, Tanreqing, Tuirejiedu, and Zhichuanling, some of which have been reported to cause hypersensitivity reactions in the clinic [40,41]. According to our research, treatment with forsythoside A or forsythoside B at an excessive dose could cause vascular leakage and elicit pseudoallergic reactions. Therefore, it is necessary to issue restrictions on the upper limit contents of forsythoside A and forsythoside B in *Fructus forsythia* used for injections. In addition, our study also proposes that the lack of limitations on the upper limit contents of some constituents in raw medicinal materials may be one of the reasons for the high incidence of ADRs in traditional Chinese medicine injections.

According to the information available in the commercial instructions, SHLI is clinically recommended for use at a dosage of 60 mg/kg, which is approximately equivalent to 600 mg/kg in mice [42]. In the previous study, we showed that treatment with SHLI at 600 mg/kg could result in endothelial dysfunction and aberrant vascular leakage in mice [16]. Therefore, the minimum vascular leakage-responsive doses used in the present study, 50 mg/kg of forsythoside A and 100 mg/kg of forsythoside B, were approximately 7 and 12 times as high as their equivalent clinical dosages (7.92 and 8.58 mg/kg), respectively. This was based on their contents of nearly 1.2% and 1.3% in the SHLI sample. Hence, there might be a series of similar active constituents that collectively lead to the pseudoallergic reactions of SHLI, which warrant further investigation. 

In recent years, forsythoside A has been reported to exhibit multiple pharmacological activities, including anti-inflammatory [43,44,45,46,47,48,49], anti-endotoxin [50,51], antivirus [52], and antipyretic [53] effects, as well as preventing hippocampal injury [54]. It is a good candidate agent for treating inflammatory diseases [46], asthma [47], Ti implant-associated infection [48], bronchitis infection [52], pyretic disease [53], and Alzheimer’s diseases [54]. In our study, we elucidated that the application of forsythoside A at a high concentration could disturb the function of the endothelial barrier and cause vascular leakiness. This shows risk in the use of forsythoside A. Therefore, a limit on the dosage of forsythoside A should be considered to prevent the occurrence of these adverse reactions.

## 4. Materials and Methods

### 4.1. Ethics Statement

This study was approved by the Research Ethics Committee of the Institute of Chinese Materia Medica, China Academy of Chinese Medical Sciences, Beijing, China (Approval Number: 20182009). All animal studies were carried out according to the recommendations of the ethical guidelines and regulations for the care and use of laboratory animals.

### 4.2. Reagents

Forsythoside A, forsythoside B, and forsythoside E were all purchased from Saibaicao Technology (Beijing, China). Rabbit polyclonal antibodies against p-MLC2 (Thr18/Ser19), MLC2, p-MYPT1 (Thr 696), and MYPT1, as well as monoclonal antibody against RhoA (67B9), were all obtained from Cell Signaling Technology (Danvers, MA, USA). Rabbit polyclonal antibody against GAPDH (FL-335) was purchased from Santa Cruz Biotechnology (Santa Cruz, CA, USA). The activated RhoA pull-down assay kit was a product of Cytoskeleton (Denver, CO, USA). FITC-dextran (MW 4×10^4^) was obtained from Sigma-Aldrich (Louis, MO, USA). Fasudil hydrochloride was provided by Chase Sun Pharmaceutical (Tianjin, China). Dulbecco’s modified Eagle’s medium (DMEM), minimum essential medium (MEM), fetal bovine serum (FBS), and the bicinchoninic acid (BCA) protein assay kit were all purchased from Thermo Fisher Scientific (Waltham, MA, USA).

### 4.3. Animals

ICR mice at 8–10 weeks of age were purchased from Vital River Laboratory Animal Technology Co., Ltd. (Beijing, China). The animals were randomly divided into designed groups based on body weight, and they were maintained under specific-pathogen-free conditions.

### 4.4. Cell Culture

Human umbilical vein endothelial cells (HUVECs) and rat basophilic leukemia-2H3 cells (RBL-2H3) were gifts from Dr. Song (Academy of Military Sciences, Beijing, China) and Dr. Qi (Chinese Academy of Medical Sciences and Peking Union Medical College, Beijing, China), respectively. HUVECs and RBL-2H3 were respectively maintained in DMEM and MEM, containing 10% fetal FBS at 37 °C in a 5% CO_2_ incubator (HERAcell, Heraeus, Hanau, Germany).

### 4.5. Assessment of Vascular Leakage in Mice 

Naive mice received a single intravenous injection (iv) of normal saline, forsythoside A, forsythoside B, or forsythoside E. Meanwhile, EB of an equal volume was given (iv) immediately after the treatment with drugs. Detailed clinical observations including changes in the skin (EB extravasation in ears), fur, as well as respiratory, digestive, nervous systems, and somatomotor activities and behavior patterns were performed for 30 min. Thirty minutes after EB administration, vascular leakage was graded according to blue stain in the ears. Based on the blue area, a score was given to each ear, where “1 to 6” represented the blue area/whole ear area at 0, 0–12.5%, 12.5–25%, 25–50%, 50–75%, and >75%, respectively. The ears of each mouse were shredded and preserved in a tube. Then, 2 mL of formamide was added for EB extraction [15,16]. The high doses of forsythoside A, forsythoside B, and forsythoside E were all designed to be given at 100 mg/kg. Doses were reduced continuously at 1/2 times until the no vascular leakage-responsive dose was found.

In the other in vivo experiment, the animals were treated with fasudil hydrochloride at 30 mg/kg once daily for three consecutive days by intraperitoneal injection (ip) to block ROCK activation. Subsequently, 100 mg/kg of forsythoside A/EB or forsythoside B/EB was administrated (iv) once at 30 min post the last treatment of fasudil. In parallel groups, the mice were given (ip) normal saline for three days and received a single administration (iv) of 100 mg/kg of forsythoside A/EB, or forsythoside B/EB. The assessment of vascular leakage was performed as described above. 

### 4.6. Histamine Assay

The mice were treated (iv) with a single dose of normal saline, forsythoside A, forsythoside B, or forsythoside E at 100 mg/kg. Blood was collected 5 min after the treatment with drugs, and plasma was obtained. The histamine concentrations were measured using commercial ELISA kits (Hermes Criterion Biotechnology, CAN) according to the manufacturer’s protocol. 

In the other experiment, the animals were pretreated with 30 mg/kg of fasudil hydrochloride for three days. Then, normal saline, forsythoside A, or forsythoside B at 100 mg/kg was injected (iv) once. In parallel groups, the mice were administered (ip) with normal saline for three days, and then received a single injection (iv) of normal saline, forsythoside A, or forsythoside B at 100 mg/kg. The collection of blood and evaluation of histamine concentration was performed as described above. 

### 4.7. Assessment of Mast Cell Degranulation

Assessment of mast cell degranulation was performed using the xCELLigence Real Time Cellular Analysis (RTCA) TP instrument (ACEA Biosciences, CA, USA) [24,25]. RBL-2H3 mast cells were seeded in E-16 plates (5×10^3^ cells/well). Then, the E-plate was loaded onto the RTCA device and the cells were cultured at 37 °C in a 5% CO_2_ incubator. The cell index (CI), which represents the cell interactions and adherent properties, was monitored every 30 min. More than 50 h after the cells were planted, at which time the curve reached the platform stage (CI stayed between 0.4 and 0.6), 5 μL of different concentrations of forsythoside A, forsythoside B, or forsythoside E were added to the cells (final concentrations at 50, 100, or 150 μg/mL). Then, the CI was recorded every 1 minute for 2 h. The high concentration, designed to be 150 μg/mL, was lower than the IC_5_ of forsythoside A, forsythoside B, and forsythoside E. The degree of mast cell degranulation was characterized by CI in this experiment [23].

### 4.8. Endothelial Permeability Assay

The endothelial permeability assay was performed in vitro by measuring the diffusion of FITC-dextran across a monolayer of confluent HUVECs. The cells were seeded on a transwell insert chamber with a pore diameter of 0.4 μm (Millicell, Merck Millipore, Cork, Ireland) and grown to confluence. The endothelial monolayer was stimulated with forsythoside A, forsythoside B, or forsythoside E (50, 100, or 150 μg/mL) for 1 h. Alternatively, it was pretreated with 10 µM of fasudil hydrochloride for 30 min and then stimulated with 150 μg/mL of forsythoside A or forsythoside B for 1 h. The high concentration, designed to be 150 μg/mL, was lower than the IC_5_ of forsythoside A, forsythoside B, and forsythoside E. In addition, it was about half of the maximum blood concentration of forsythoside A, based on the results presented in a pharmacokinetic study of rats treated with forsythoside A at 50 mg/kg (approximately equivalent to mice treated with forsythoside A at 100 mg/kg) [55]. Following incubation, all drugs were removed. Then, 300 µL of DMEM containing FITC-dextran (1 mg/mL) and 400 µL of blank DMEM were added to the upper compartment and lower compartment of the transwell insert chambers, respectively. Samples of FITC-dextran that diffused across the endothelial monolayer were collected from the lower compartment after 10, 20, 30, 40, 50, and 60 min, respectively. Fluorescence was determined immediately using a microplate reader (Thermo Scientific Varioskan Flash, Thermo Fisher Scientific, MA, USA) with an excitation wavelength of 490 nm and an emission wavelength of 520 nm. The fluorescent apparent permeability coefficient (P_app_, cm/s) was calculated according to the equation described in Reference [16].

### 4.9. Preparation of Cell Lysates

HUVECs were incubated with forsythoside A, forsythoside B, or forsythoside E at 50, 100, or 150 μg/mL for 30 min. Alternatively, they were pretreated with 10 µM of fasudil hydrochloride for 30 min and then stimulated with 150 μg/mL of forsythoside A or forsythoside B for 30 min. Cells were lysed in cold commercial RIPA buffer supplemented with protease and phosphatase inhibitors and then centrifuged at 12,000 rpm for 10 min at 4 °C. The supernatants of lysates were collected. The total protein concentration was determined using the BCA protein assay kit following the provided protocol. GTP-RhoA was obtained using a commercial pull-down assay kit according to the manufacturer’s instructions. 

### 4.10. Immunoblot Analysis

Cell homogenates with equal total protein (50 μg) were loaded in each lane of SDS-polyacrylamide gel electrophoresis (SDS-PAGE, 8–12%). The separated proteins were subsequently transferred onto polyvinylidene fluoride (PVDF) membranes and blocked with blocking buffer at room temperature for 1 h, then blotted with indicated primary antibodies overnight at 4 °C. After washing with Tris-buffered saline with Tween-20 (TBST), bound primary antibodies were detected with a secondary antibody for 2 h at room temperature. The bands were washed with TBST and probed using a chemiluminescence detection kit.

### 4.11. Molecular Docking

Auto Dock Vina was used for molecular docking and for calculating the binding score [56]. The GTP-RhoA analog complex was downloaded from the RCSB Protein Data Bank (PDB) using PDB ID: 3TVD [57]. Auto Dock Tool prepared this protein, and the active sites were predicted using the PyMOL Tool. The structures of compounds (forsythoside A, forsythoside B, forsythoside E) were drawn using Chem Draw Professional 15.0, and the structures were transferred to Chem3D. Then, the energy minimization of the structures’ MM2 force field was calculated, and the minimized ligands in PDB format were saved as PDBQT files using the Auto Dock Tool. The binding site of GTP-RhoA with a grid center was at x = 17.083, y = 23.222, z = 24.306. The number of points in each dimension was designed as X = 20, Y = 20, Z = 20, and spacing (Å) = 1.0. The interactions between GTP-RhoA and the compounds were visualized and analyzed using the PyMOL Visualization Tool.

### 4.12. Statistical Analysis

All analyses were performed using SPSS 16.0 software. The results are described as the mean ± standard error of the mean (M ± SEM). Quantitative data among multiple groups were analyzed by one-way analysis of variance (ANOVA). The Rank-test analyzed the score of blue stain in the ears. *p* < 0.05 was considered to represent a statistically significant difference.

## 5. Conclusions

In conclusion, forsythoside A and forsythoside B contribute to Shuanghuanglian injection-induced pseudoallergic reactions through the RhoA/ROCK signaling pathway.

## Supplementary Materials

The following are available online at https://www.mdpi.com/1422-0067/20/24/6266/s1, Figure S1: Contents of forsythoside A, forsythoside B, and forsythoside E in Shuanghuanglian injection.
