# Supplementary material for: Forsythoside A and Forsythoside B Contribute to Shuanghuanglian Injection-Induced Pseudoallergic Reactions through the RhoA/ROCK Signaling Pathway
Source: Int J Mol Sci. 2019 Dec 12;20(24):6266. doi: 10.3390/ijms20246266 (PMC6940901; doi:10.3390/ijms20246266)
Supplement: Supplementary file 1 [file ijms-20-06266-s001.pdf]

RESULTS

Contents of Forsythoside A, Forsythoside B, and Forsythoside E in Shuanghuanglian Injection

Forsythoside A, forsythoside B, and forsythoside E were all detected in the tested SHLI sample. Their contents were 1.2%, 1.3% and 1.5%, respectively. The linear ranges of forsythoside A, forsythoside B, and forsythoside E were 13.28-425.00, 22.28-712.50, and 10.74-343.75 ug/mL, respectively. The linear correlation coefficient of forsythoside A, forsythoside B, and forsythoside E were 0.9998, 0.9995, and 0.9996, respectively.

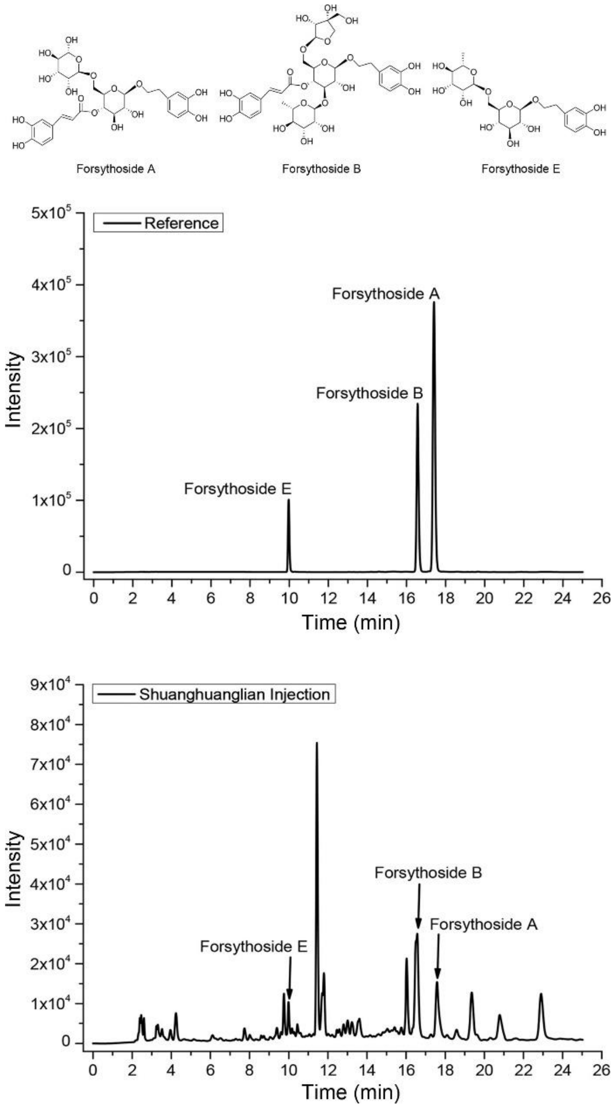

Figure S1. Contents of forsythoside A, forsythoside B, and forsythoside E in Shuanghuanglian injection.

MATERIALS AND METHODS

Analysis of Forsythoside A, Forsythoside B, and Forsythoside E in Shuanghuanglian Injection

The contents of forsythoside A, forsythoside B, and forsythoside E in SHLI were measured on a Shimadzu HPLC system (DGU-20A5, Kyoto, Japan). Components were separated on an Agilent column (ZORBAX Eclipse XDB-C18, 250 × 4.6 mm, 5 μm) at 30 °C with a flow rate of 1 mL/min. Injection volume was 5 μL. The mobile phase was composed of water with 1% formic acid (solvent A) and acetonitrile (solvent B), with the following gradient: 5-10% B during 0-5 min, 15-20% B during 5-10 min, 20-22% B during 10-20 min, 5% B during 20-25 min. The UV spectrum was recorded in 275 nm.
